# Occult Hepatitis B Virus Infection in Nigerian Blood Donors and Hepatitis B Virus Transmission Risks

**DOI:** 10.1371/journal.pone.0131912

**Published:** 2015-07-06

**Authors:** Opaleye O. Oluyinka, Hoang Van Tong, Sy Bui Tien, Ademola H. Fagbami, Olusegun Adekanle, Olusola Ojurongbe, C.-Thomas Bock, Peter G. Kremsner, Thirumalaisamy P. Velavan

**Affiliations:** 1 Institute of Tropical Medicine, University of Tübingen, Tübingen, Germany; 2 Deparment of Medical Microbiology and Parasitology, Ladoke Akintola University of Technology, Ogbomosho, Nigeria; 3 Department of Infectious Diseases, Robert Koch Institute, Berlin, Germany; 4 Obafemi Awolowo University, Ile Ife, Nigeria; 5 Department of Molecular Pathology, University of Tübingen, Tübingen, Germany; 6 Fondation Congolaise pour la Recherche Medicale, Brazzaville, Republic of Congo; 7 Department of Molecular Biology, 108 Military Central Hospital, Hanoi, Vietnam; CEA, FRANCE

## Abstract

**Background:**

Occult hepatitis B virus infection (OBI) characterized by the absence of detectable HBsAg remains a potential threat in blood safety. We investigated the actual prevalence, viral factors and genotype of OBI infections in Nigerian blood donors.

**Methods:**

Serum collected from two blood banks were reconfirmed as HBsAg seronegative by ELISA. Forty HBsAg positive samples were employed as controls. HBV-DNA was amplified from all donors and viral loads were determined using quantitative real-time PCR. Antibodies to the HBV core, surface and HBe antigen (anti-HBc,anti-HBs,HBeAg) were measured. The *PreS/S* and *PreC/C* regions of the HBV genome were sequenced.

**Results:**

Of the 429 blood donors, 72(17%) were confirmed as OBI by DNA detection in different reference labs and excluded the concern of possible contamination. Of the 72 OBI samples, 48(67%) were positive for anti-HBc, 25(35%) positive for anti-HBs, and 2(3%) positive for HBeAg. Of the 72 OBI samples, 31(43%) were seropositive for either anti-HBc, anti-HBs or HBeAg, 21 (30%) positive for both anti-HBc and anti-HBs,one positive for both anti-HBc and HBeAg. None of the OBI samples were positive for all three serological markers. The viral load was <50copies/ml in the OBI samples and genotype E was predominant. The L217R polymorphism in the reverse transcriptase domain of the HBV polymerase gene was observed significantly higher in OBI compared with HBsAg positive individuals (*P*<0.0001).

**Conclusion:**

High incidence of OBI is relevant in high endemic areas worldwide and is a general burden in blood safety. This study signifies the high prevalence of OBI and proposes blood donor samples in Nigeria should be pre-tested for OBI by nucleic acid testing (NAT) and/or anti-HBc prior to transfusion to minimize the HBV infection risk.

## Introduction

Human hepatitis B virus (HBV) infection remains the primary cause of liver cirrhosis and hepatocellular carcinoma (HCC), which are major contributors to global mortality [1]. The concurrent presence of hepatitis B surface antigen (HBsAg) and HBs antibody are observed in both acute and chronic hepatitis B infection. Chronic hepatitis B is clinically defined as the frequent detection of HBsAg for at least six months after acute infection [2]. In addition, chronically infected patients potentially have high levels of the hepatitis B envelope antigen (HBeAg) in their serum. Chronic HBV carriers are clinically defined by the presence of HBsAg and anti-HBc, as HBV produce the core antigen (HBcAg) during an active replication phase[3]. Altogether, the active chronic state is associated with high levels of HBV-DNA, liver inflammation, elevated liver enzymes and the highest risk of cirrhosis and HCC [2]. Serological markers that detect HBV antigens and antibodies are often used to determine the stages of HBV infection. Utilizing these markers, studies have revealed that HBV is cleared after the resolution of this acute infection; however, in approximately 5% to 10% of adults and in 80–90% of neonates a chronic carrier state still may persist [1,3,4].

Occult hepatitis B virus infection (OBI) has been described for decades, and Nucleic Acid Testing (NAT) for HBV-DNA detection has confirmedthe existence of the OBI, which isdefined as the presence of HBV-DNA in the absence of detectable HBsAg with or without anti-HBV antibodies[5]. This phenomenon is becoming increasingly recognized in several clinical settings worldwide [6]. Studies on a large set of blood donors using NAT confirmed this phenomenon of OBI and formed the basis of mandatory NAT for transfused blood units in many developed countries [7,8]. Such a testing regimen has not been incorporated into the testing algorithms of many laboratories in developing countries including Nigeria. Studies on OBI prevalence in Nigeria is unclear, with one single study that utilized a smaller sample size (n = 28) reported no prevalence of OBI in healthy subjects [9].The prevalence of OBI in blood donors has been confirmed from different geographic areas and ranges from less than 1% to 16% depending on the endemicity of HBV infection [10–13] In a general Korean adult population, the prevalence of OBI was 0.7% [14] and the prevalence of OBI was 18% and 8% in resolved HBV infection group and in HBV seronegative individuals (negative for HBsAg, anti-HBs and anti-HBc), respectively [15]. However, the prevalence of OBI was 64% in liver transplanted patients, 62% in HCC patients, 27% in hemodialysis patients and up to 45% in HCV and HIV infected patients [16,17].

In the current study we investigated the actual prevalence, viral factors and genotypes of occult HBV infections in Nigerian blood units prescreened negative for HBsAg. This study emphasizes the use of NAT for screening HBV before blood transfusion.

## Materials and Methods

### Ethics statement

Informed written consent was obtained from all the blood donors. The study was approved by the ethics committee of Ladoke Akintola University of Technology Teaching Hospital, Osun, Nigeria.

### Sampling

Four hundred and twenty-nine (n = 429) serum samples were randomly collected from blood donors representing two major blood banks in southwestern Nigeria (male/female: 202/227). As a routine practice, these two blood banks screen for HBsAg using a commercial rapid test (Clinotech Diagnostics and Pharmaceuticals, Inc. Canada) and have excluded the possibility of a HBV infection. The sensitivity and specificity of the rapid test were> 99% and 97.0%, respectively. Also allHBsAg negative blood donors were prescreened for HIV status by ELISA in respective blood banks and were determined HIV negative. In addition, forty (n = 40) HBsAg positive serum samples identified as carriers were included as controls. Viral DNA was extracted from 200μL of serum samples using commercially available QIAampDNA Blood Mini kit (Qiagen GmbH, Hilden, Germany) following the manufacturer instructions and was stored at -20°C until use.

### Serology

All 429 samples were reanalyzed and reconfirmed for their HBsAg seronegative status by ELISA (Human diagnostics, Wiesbaden Germany). Those samples that were HBsAg seronegative and positive for HBV DNA were subsequently tested for anti-HBc, anti-HBs, HBeAg and anti-HCV using ELISA (Human diagnostics, Wiesbaden, Germany; and DRG Diagnostics, Marburg, Germany) following the manufacturer’s instructions.

### Detection of HBV-DNA

The presence of HBV-DNA was examined in all samples using a routine diagnostic PCR in the reference labs. Primer pairs were designed from the highly conserved overlapping regions of the *S* and *P* regions of the HBV genome. A nested PCR was performed: Outer primer pairs were HBPr134 (sense) 5’-TGCTGCTATGCCTCATCTTC-3’ and HBPr135 (antisense) 5’-CAGAGACAAAAGAAAATTGG-3´ and the inner primers were HBPr75 (sense) 5’-CAAGGTTATGTTGCCCGTTTGTCC-3’ and HBPr94 (antisense) 5’- GGTATAAAGGGACTCACGATG-3’. PCR amplifications were carried out in 25μl reaction volumes with 5ng of genomic DNA, 10x PCR buffer (20mM Tris-HCl pH 8.4, 50 mMKCl; Qiagen), 2mM of dNTPs, 50ng of each primer and 1U AmpliTaq gold DNA polymerase (Applied Biosystems) on a PTC 200 cycler (Peltier Thermal cycler Watertown, Massachusetts, USA). Thermal cycling parameters were: initial denaturation at 94°C for 2 min, followed by 35 cycles of 30sec at 94°C denaturation, 30 sec at 52°C annealing temperature, 45 sec at 72°C extension, followed by a final extension of 5 min at 72°C. Thermal cycling parameters remained the same as in the first PCR round except for the number of cycles which were increased to 40 cycles in the subsequent amplification. A positive control (HBV plasmid DNA) and a negative control of the master mix were integrated to each run to validate the PCR products that produce a 340bp fragment. The detection limit of the HBV DNA by nested PCR is approximately 2.5 copies per reaction (between30-40copies/mL). All 72 PCR-positive samples representing OBI and thirty (n = 30) PCR-positive samples from HBsAg positive carriers were successfully sequenced after purification of the nested PCR product using GFX PCR purification kit (Healthcare, Buckinghamshire, UK) according to the manufacturers’ instruction. Sequencing was performed using the BD Terminator cycle sequencing kit and analyzed on ABI PRISM Genetic analyzer 3130XL (Applied Biosystems, CA) according to manufacturer’s instructions. The sequences were analyzed by using BioEdit 9.7 and Codon-code Aligner 4.0 software.

### Independent re-confirmation of HBV-DNA detection in referral centre

The samples those positive for HBV-DNA were reconfirmed independently at a different laboratory at the Division of Viral Gastroenteritis and Hepatitis Pathogens and Enteroviruses, Robert Koch Institute, Berlin by nested PCR with different primer pairs and by subsequent sequencing of the *preS/S* and *preC/C* regions. The nested PCR for the *preS/S* region was performed using sense primer HBPr134 as described above and two antisense primers (HBPr135: 5’-CAGAGACAAAAGAAAATTGG-3’ and HBV-66: 5’-CACAGATAACAAAAAATTGG-3’) for the first PCR round. Primers utilized for subsequent nested PCR were HBV-24 (sense) 5’-CAAGGTATGTTGCCCGTTTGTCCT-3’ and two antisense primers (HBV-64: 5’-GGACTCAMGATGYTGCACAG-3’ and HBV-41: 5’-GGACTCAMGATGYTGTACAG-3’) that amplified a 318bp fragment. PCR was carried out in a 12.5μl reaction volume containing 5μl of DNA, 0.4μM of each primer and 6.25μl of Hot start Master Mix (Qiagen). Thermal cycling parameters were initial denaturation at 95°C for 15 min, followed by 35 cycles (30 cycles for the second round) of 30 sec at 94°C denaturation, 30 sec at 55°C annealing temperature (50°C for the second round), 1min at 72°C extension (30 sec for the second round), followed by a final extension of 10 min at 72°C (5 min for the second round). A positive (HBV plasmid DNA) and a negative control were integrated to each run to validate the PCR products. Each sample was tested at least three times and a triplicate nested PCR was employed to reconfirm the results. The nested PCR was evaluated for detecting and genotyping HBV with WHO panels. Sample processing (DNA/RNA-extraction, template and master-mix preparation) and PCR were performed in separate and dedicated laboratory rooms that are certified for molecular diagnostics using standard precautions to prevent assay contamination.

Furthermore, the *preC/C* region was sequenced to investigate the diversity of Nigerian HBV isolates.Briefly, semi-nested PCR was used to amplify a 655bp fragment in the *preC/C* region with sense primers HBV-102: 5’-GGAGACCACCGTGAACGC-3’ (for the first round) and HBV-101: 5’-CTGGGAGGAGTTGGGGGA-3’ (for the second round). The antisense primer HBV-99: 5’-TTCTTCTTCTAGGGGACCTGCCTCAGTCC-3’ (for genotype A) and HBV-100: 5’-TTCTTCTTCTAGGGGACCTGCCTCATCGT-3’ (for genotype non-A) were used for both first and second PCR rounds in separate reactions for each sample. The PCR products from the second round were cleaned up by using Exo SAP-IT kit (USB, Affymetrix, USA). Sequencing was performed with 1–5μl of purified PCR product, 1μl of BigDye and 5μM of primer. The sequences were analyzed by using BioEdit 9.7 and Genious Pro 5.4.3.

### Quantification of HBV-DNA

Quantification of HBV-DNA was performed with quantitative real-time PCR in a 7300 Real-Time PCR System (Applied Biosystems, Perkin-Elmer, Foster City, CA). HBV-plasmid DNA was used to generate a standard curve following a serial 10-fold dilution. Our quantitative HBV-specific PCR assays were routinely standardized using the WHO standard (NIBSC code: 97/750). In addition, the detection of HBV-DNA by real-time PCR in our laboratories was validated by Quality Control for Molecular Diagnostics (QMCD, which is an independent International External Quality Assessment (EQA) / Proficiency Testing (PT) organization) round robin tests.

### Statistical and phylogenetic analysis

All analysis was performed using the SPSS v15 software. Categorical variables were compared using Fisher’s exact test. The significance was set at a *P*-value of less than 0.05. Sequencing results were analyzed using BioEdit 9.7 software and Geneious Pro (Version 5.5.7, Biomatters Ltd, Auckland, New Zealand). HBV genotypes were determined using phylogenetic analysis and the phylogenetic tree reconstruction was carried out by MEGA 5 software. The evolutionary history was inferred using the Neighbor-Joining method andthe evolutionary distances were computed using the Maximum Composite Likelihood method. The respective sequences representing the *preS/S*(KT001260-KT001331) and the *preC/C* regions (KT001332-KT001381) were submitted to NCBI Genbankdatabase. For alignment and HBV-genotyping, eight prototype HBV-genotype sequences retrieved from the NCBI Genbank were used: Genotypes A (U87742, M57663), B (D50522, D23678), C (D23680, M38636), D (AF151735, X02496), E (X75657, AB032431, AB091256), F (AY090455, X69798), G (AB064313, AF160501) and H (AY090454, AB059659).

## Results

### Serological characterization of OBI in Nigeria

All 429 serum samples still remained negative for HBsAg when retested by a second ELISA in another lab. Nevertheless, 72 (17%) individuals were HBV-DNA positive by a nested PCR using HBV specific primer pairs. The detection of HBV-DNA in these 72 donors were independently validated in another institute with different primer pairs and subsequent sequencing for both *preS/S* and *preC/C* regions in the HBV genome. Of these 72 positive HBV-DNA samples, HBV serological markers were assayed. Serological testing of all 72 OBI samples revealed 48 (67%) positive samples for anti-HBc, 25 (35%) remained positive for anti-HBs and 2 (3%) had detectable HBeAg. Of the 72 OBI samples investigated, 31(43%) were seropositive for either anti-HBc, anti-HBs or HBeAg. In addition, 2 (3%) of the 72 with OBI were positive for anti-HCV, 21 (29%) of the 72 samples were positive for both anti-HBc and anti-HBs, 27 (38%) samples were positive for anti-HBcand negative for anti-HBs, 4 (6%) were positive for anti-HBs and negative for anti-HBc while 20 (28%) samples had no detectable antibodies to neither HBsAg nor HBcAg (Table 1). Quantified HBV viral load ranged from unquantifiable to 58 copies/ml in all the OBI samples, of which only 17 (24%) of the 72 OBI samples were quantifiable with a mean of 14.4 copies/ml. The majority of the OBI carriers were females (44/72). The highest prevalence of the OBI was in the age group of 30–39 years. Of the investigated 40 HBsAg positive controls, all individuals remained positive for anti-HBc and 9 (23%) were HBeAg positive (Table 1).

**Table 1:** Baseline characteristics of occult hepatitis B and HBsAg positive patients.

Characteristics	Occult HBV (n = 72)	HBsAg positive (n = 40)	*P* value
Mean Age (Years)	40.2 ± 13.9	36.8 ± 8.7	NS
Sex (Male/Female)	28/44	21/19	NS
Mean Viral load (copies/ml)	5.7	54	< 0.0001
HBeAg positive	2 (2.8%)	9 (23%)	< 0.0001
anti-HBc positive	48 (66.7%)	40 (100%)	< 0.0001
anti-HBs positive	25 (34.7%)	0	< 0.0001
anti-HBcand anti-HBs positive	21 (29.2%)	0	< 0.0001
anti-HBcandHBeAg positive	1 (1.4%)	9 (23%)	< 0.0001

### Molecular and genetic characterization of OBI isolates

**Fig 1:**
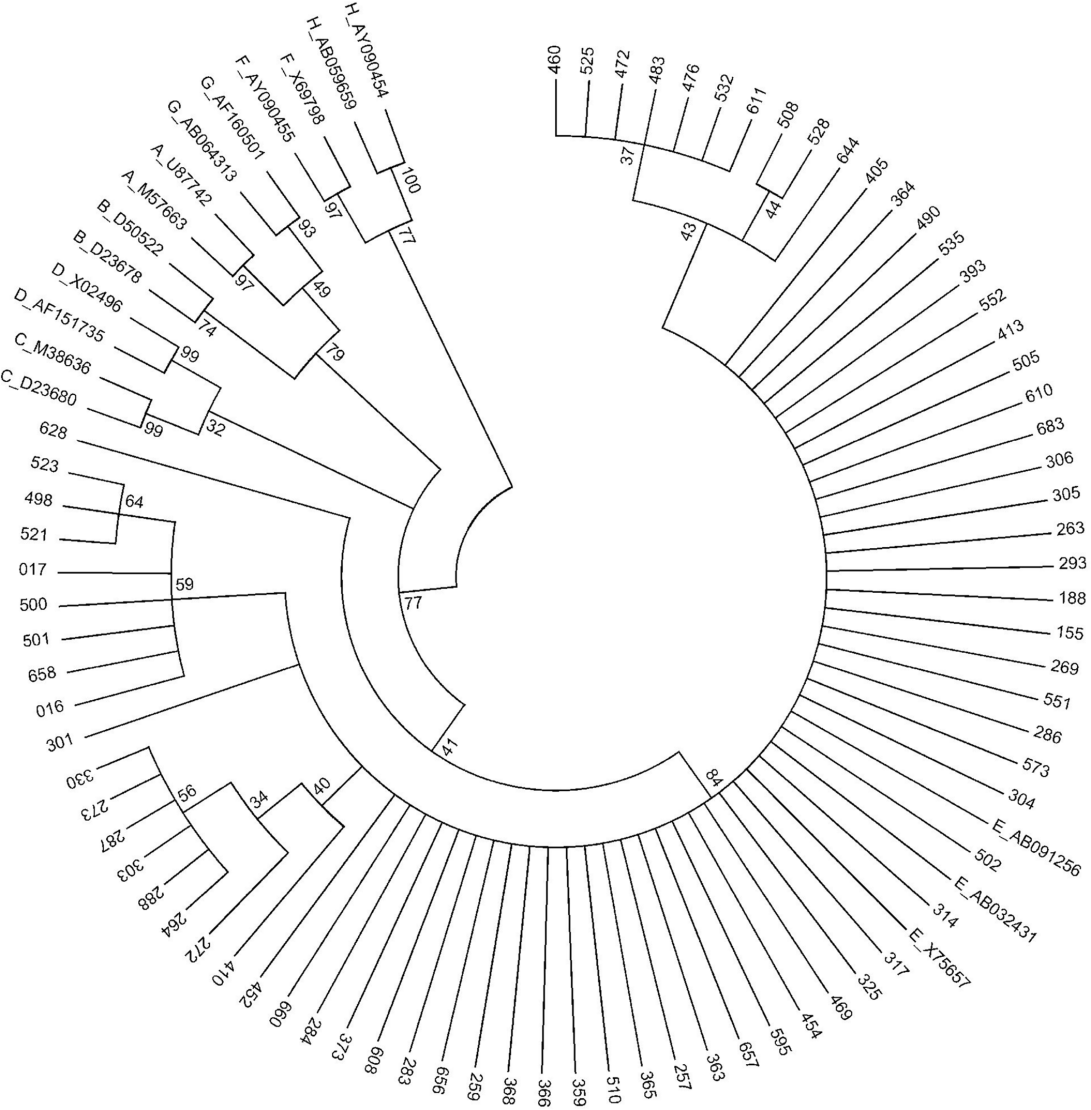
Reconstructed phylogenetic tree of *preS/S* region of the HBV genome. Phylogenetic analysis inferred from distance analysis (Kimura 2 parameters model) and neighbor-joining reconstruction from *preS/S* region of OBI sample sequences showing that the HBV sequences clustered in the HBV genotype E branch. HBV sequences are referred to as “number”, i.e., “016”. The HBV sequences were compared to HBV reference sequences gathering the 8 HBV genotypes (NCBI-Genbank accession numbers are denoted). The numbers at the nodes indicate bootstrapping values in percentage of 1000 replicates.

The *preS/S* region of 72 OBI samples were sequenced and phylogenetic analysis revealed that all OBI samples belong to the HBV genotype E (Fig 1). Of the additional 30 HBsAg positive individuals investigated, HBV genotype E and A were observed in 28 (93%) and 2 (7%) individuals, respectively. In addition, the phylogenetic analysis also showed that 2 HBsAg positive samples were HBV sub-genotype A2. Investigation of the RT domain of the HBV polymerase gene of the 72 OBI individuals revealed a number of non synonymous substitutions. The G779 nucleotide substitution that translates L217R amino acid in the RT domain of the polymerase gene was found significantly higher (99%) in OBI compared to HBsAg positive samples (47%) included in this study (*P*<0.0001) (Table 2). Other mutations were observed in the OBI sequence of the RT domain including L144F, P177R, L179R and K212R substitutions. In addition, the G779 nucleotide substitution also resulted in amino acid change at L209V in the *S* gene. Consequently, the L209V amino acid substitution was significantly higher in OBI (99%) compared to HBsAg positive samples. Also, the amino acid substitutions R169G and SN204G were observed frequently in OBI (4% and 8%, respectively) but not in HBsAg positive samples. Other OBI specific amino acid substitutions observed were A128V, E164G, S171A and P188L. Of those, the substitutions A128V, E164G are in the major hydrophilic region (MHR) of the *S* gene (Table 3).

**Table 2:** Mutations in the RT domain of the *P* gene in occult hepatitis B and HBsAg positive patients.

Amino acid substitution	OBI Group (n = 72)	HBsAg positive (n = 30)
T128A	0	1 (3%)
D205A	0	1 (3%)
L217R	58 (80.6%)	10 (33%)
K212R + V214A	0	1 (3%)
H135T + L217R	0	1 (3%)
L144F + L217R	1 (1.4%)	0
I163V + L217R	0	2 (7%)
P177R + L217R	3 (4.2%)	0
L179R + L217R	1 (1.4%)	0
K212R + L217R	8 (11.1%)	1 (3%)
Wild type	1 (1.4%)	13 (43%)

**Table 3:** Mutations in the *S* gene in occult hepatitis B and HBsAg positive patients.

Amino acid substitution	OBI Group (n = 72)	HBsAg positive (n = 30)
TN131P	0	1 (3%)
T189I	0	1 (3%)
M197D	0	1 (3%)
L209V	56 (79.2%)	10 (33%)
A128V + L209V	1 (1.4%)	0
S154L + L209V	0	2 (7%)
E164G + L209V	1 (1.4%)	0
R169G + L209V	3 (4.2%)	0
S171A + L209V	1 (1.4%)	0
P188L + L209V	1 (1.4%)	0
SN204G + CY206H	0	1 (3%)
SN204G + L209V	8 (11.1%)	1 (3%)
P1358H + S154L + Y200S	0	1 (3%)
Wild type	1	12 (40%)

**Fig 2:**
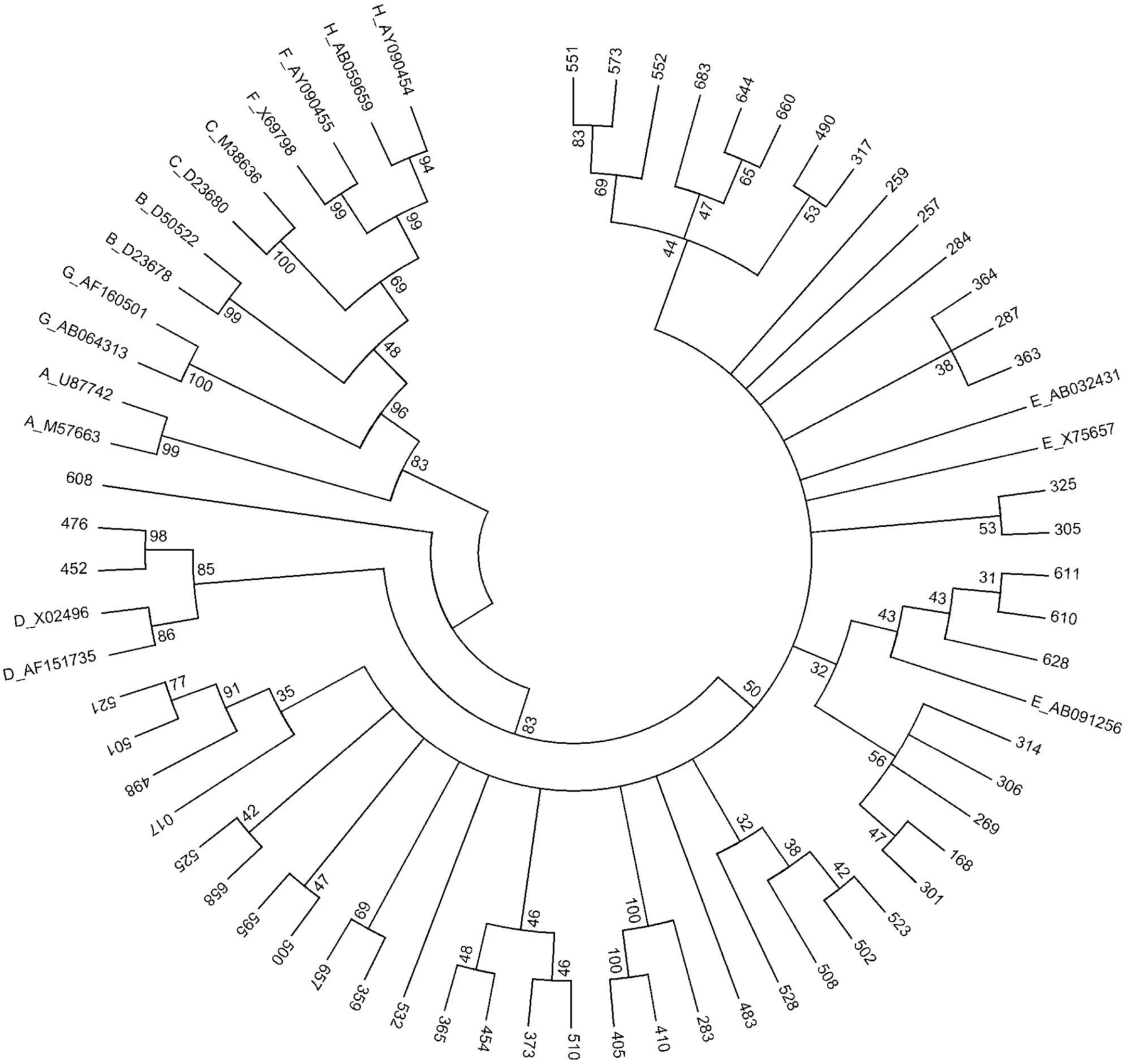
Reconstructed phylogenetic tree of *preC/C* region in the HBV genome. Phylogenetic analysis inferred from distance analysis (Kimura 2 parameters model) and neighbor-joining reconstruction from *preC/C* region of OBI sample sequences showing that the HBV sequences mostly clustered in the HBV genotype E branch. HBV sequences are referred to as “number”, i.e., “017”. The HBV sequences were compared to HBV reference sequences gathering the 8 HBV genotypes (NCBI-Genbank accession numbers are denoted). The numbers at the nodes indicate bootstrapping values in percentage of 1000 replicates.

In addition, 50 of the 72 OBI samples were sequenced for *preC/C* region and a phylogenetic tree was reconstructed (Fig 2). The nucleotide diversity in *preS/S* and *preC/C* regions of OBI samples demonstrated that the samples were specific isolates and excludes the probability of contaminants. The phylogenetic tree of *preC/C* corroborated 47 OBI samples belonged to HBV genotype E. In addition, the *preC/C* phylogenetic tree showed the similarity between HBV genotype E and D in this region by classifying all OBI samples. HBV genotype E and D in a group with 84% bootstrap value supported. Two of the three non HBV genotype E OBI samples (sample 452 and 476) belong to HBV genotype D with 86% bootstrap iterations. Sample 608 has its own clade separated from HBV genotype D and E branch. Interestingly, 50 of the *preC/C*sequences revealed that four OBI strains carry the *PreC* stop codon W28* mutant at position 1896.

## Discussions

High prevalence of OBI is relevant in high and low endemic areas worldwide and OBI is a general burden with regard to blood donors. In this study, we investigated the high incidence of OBI among Nigerian blood donors by NAT and characterized the serological as well as molecular and genetic aspects of those OBI strains in different lab settings. Our results strongly justify that NAT remains a prerequisite for avoidance of HBV transmission risks in the investigated country.

The prevalence of OBI varies to a great extent in different countries, depending on a number of factors that includes HBV endemicity, liver disease, HBV screening method and primers employed for NAT. In NortheastChina, the prevalence of OBI was observed up to 10.6% among 359 HBsAg-negative healthy individuals[18]. In other studies, only 0.1% of OBI was detected among 10,727 seronegative blood donors from Taiwan [19] and 3% in an Italian migrant population [20]. Recently in Laos a HBV endemic region reported high OBI prevalence (10.9%) among blood donors who were HBsAg-negative, anti-HBc and/or anti-HBs-positive [21]. In North Africa, a study conducted among 1026 Egyptian blood donor samples revealed that 8% were reactive to anti-HBc and 0.5% were positive for HBV-DNA [22]. The data on OBI prevalence is limited in sub-Saharan Africa. Nevertheless, studies in patients with HIV infection from Ivory Coast and Sudan have shown that OBI prevalence was 10% and 15%, respectively[23,24]. Recently, OBI has been shown to be 8% among 100 repeat blood donors in South-Eastern Nigeria [25]. OBI is unexpectedly high in Nigeria taking into account that the seroprevalence of HBV in Nigeria is from 9%-39% [26]. The prevalence of 17% reported in current study is rather high but this might not be surprising for a sub-Saharan country endemic to HBV where the prevalence of anti-HBc exceeds over 50% of the blood donor population [27]. In addition, our results also showed the evidence of previous infection with HCV (11% of OBI positive with anti-HCV). In the high risk groups of OBI (e.g. HCV co-infection), the OBI prevalence was up to 64% [16],therefore the possible reasons for the high prevalence of OBI could be due to the high prevalence of HBV infection in Nigeria, and the blood donors who previously infected with other infectious diseases are high risk groups of OBI. Our study demonstrates that the OBI samples had significantly lower HBV-DNA copies compared toHBsAg positive patients. The low level of viral load inferred in this current study corroborate a previous report, that showed almost all OBI cases are infected with replication competent HBV, revealing a strong suppression of replication activity and gene expression, thereby resulting in a reduced viral load [28].

Understanding the molecular and immunological mechanisms in determining an OBI has to be investigated explicitly. A hypothesis proposed for the development of OBI was that mutations in the *PreS/S* regions may alter HBsAg antigenicity thereby inhibiting anti-HBs production. A single mutation at the ‘‘a” determinant (amino acids 124–147, e.g., G145R) of HBsAg can lead to a change in the immunologic epitope thus inhibiting HBsAg secretion. The amino acid substitutions in the RT domain of the *P* gene contribute to low copies of HBV-DNA and HBsAg synthesis that may have an associative effect on an occult infection. In our study, all of the OBI strains had the G779 nucleotide substitution that results in amino acid change at L217R in the overlapping RT domain of the *P* gene and this may account for the occult nature of the HBV infection in our study. Another study also observed that the L217R substitution within the RT domain was predominant in OBI individuals [29]. In addition, mutations in the major hydrophilic region (MHR) also influence the antigenicity and can impair virion secretion consequently leading to HBsAg detection failure in OBI individuals [12]. However, the L209V substitution, which corresponds to the L217R substitution, is not located at the ‘‘a” determinant or in the MHR of the *S* gene. Therefore, we assume that the L217R mutation may possiblybe associated with OBI status in investigated samples. However, the association of this L217R substitution with OBI has to be validated from further studies. In addition, we also observed that the substitutions A128V and R169G (located in the MHR of the *S* gene) occurred only in OBI samples and these maybe the escape mutations during OBI development. Furthermore, in agreement with a previous study from Ghana [30], we observed the *PreC* stop codon W28* mutant at position 1896 in four of the investigated OBI strains. The mutation in the stop codon was shown to associate with the HBeAg status [31]. Interestingly, this mutation has also proposed to be associated with the OBI status [32,33]. However, more studies are needed to warrant the role of W28* (G1896A) mutation in OBI development.

HBV genotype E is the most prevalent in Nigeria [34] that was in accordance with our tested OBI individuals. HBV genotype E is endemic in West Africa and also exhibits low genetic diversity [35]. Our study revealed no difference in HBV genotypes between occult HBV-infected and HBsAg-positive individuals with HBV genotype E being the predominant genotype in both groups. In addition, the HBV sub-genotype A2, which was previously reported in South Africa [36], were observed in our study samples from two Nigerian individuals positive for HBsAg. This finding contradictsa previous study and indicates that the HBV subgenotype A3 and the recombination between HBV genotypes A and E were observed frequently in West Africa [37,38]. In this current study, we could not genetically distinguish between the HBV genotypes E and D of two OBI isolates by phylogenetic analysis of the *PreS/S* and *PreC/C* regions. Therefore, genotyping and sub-genotyping of HBV should be identified based on the analysis of full length sequences. However, full length HBV sequences are not always available so that phylogenetic analyses of the *PreS/S* regions are widely accepted for routine HBV genotyping. Therefore, based on the phylogenetic analyses of the *PreS/S* regions, we assumed that all our OBI samples belong to HBV genotype E.

Those OBI individuals positive for anti-HBc, anti-HBs and HBV-DNA represent the viral persistence after recovery with a low viral load as seen in this study as also observed in previous reports [27,39]. A plausible explanation for this observation is that anti-HBs antibody is poorly neutralizing due to loss of recognition, allowing these mutant viruses to escape neutralization even when antibody is present at protective levels [10,40]. The OBI individuals positive only for HBV-DNA without any detectable HBV antibodies might be as a result of long lasting persistence of HBV cccDNA or the possibility of integration of the HBV-DNA into the host genome [6]. The detection of HBeAg without detectable HBsAg in two samples suggests the presence of wild type variants in a lower percentage as demonstrated previously [30]. A limitation of our study is that there was lack of anti-HBc data in the blood donors negative for both HBsAg and HBV-DNA. Nevertheless, in agreement with a previous study validating the inclusion of anti-HBc testing in Egyptian blood donors negative for HBsAg[22], we assume that the individuals who are negative for both HBsAg and HBV-DNA but positive for anti-HBc might have been infected with HBV but the HBV-DNA viral loads are below detection level. Therefore, anti-HBc should be considered as an additional serological marker for screening of HBV infection in blood donors. The anti-HBc positive donors remained positive for HBV-DNA to a greater extent and the donors who are positive with anti-HBc but negative with HBV-DNA should also be excluded for blood transfusion to avoid the risk of HBV transmission. This is well in agreement with a report that revealed anti-HBc positivity alone contributes to OBI with low viral replication [8]. The viral DNA may persist either ascccDNA or may integrateinto the host genome in hepatocytes, thus HBV is undetectable in the serum [6]. However,viral DNA can possibly be detected in liver tissue of healthy individuals positive for anti-HBc[41,42]. Individuals who were positive for anti-HBC alone had low OBI occurrence (1.7%) in a study reported from South Korea. The lower OBI occurrence in anti-HBC alone may be because of low viral load[43].

In conclusion, this study suggests the residual risk of HBV transmission from Nigerian blood donors and can be minimized by employing NAT assays. Hence, the introduction of HBV-DNAassays will be a useful tool in the quest to approach near to zerorisk of HBV transmission. We therefore propose that blood donor samples in Nigeria should be tested for OBI status by NAT and/or at least by anti-HBc screening prior to transfusion that minimizes the risk of acquiring HBV burden in transfused individuals. Our study also suggests that the mutation in the RT domain of the *P* gene and the MHR of the *S* gene may account for the occult nature of the HBV infection.
